# Liquid metal amoeba with spontaneous pseudopodia formation and motion capability

**DOI:** 10.1038/s41598-017-07678-8

**Published:** 2017-08-03

**Authors:** Liang Hu, Bin Yuan, Jing Liu

**Affiliations:** 1 Beijing Key Lab of Cryo-Biomedical Engineering and Key Lab of Cryogenics, Technical Institute of Physics and Chemistry, Chinese Academy of Sciences, Beijing, 100190 China; 2 Department of Biomedical Engineering, School of Medicine, Tsinghua University, Beijing, 100084 China

## Abstract

The unique motion of amoeba with a deformable body has long been an intriguing issue in scientific fields ranging from physics, bionics to mechanics. So far, most of the currently available artificial machines are still hard to achieve the complicated amoeba-like behaviors including stretching pseudopodia. Here through introducing a multi-materials system, we discovered a group of very unusual biomimetic amoeba-like behaviors of self-fueled liquid gallium alloy on the graphite surface immersed in alkaline solution. The underlying mechanisms were discovered to be the surface tension variations across the liquid metal droplet through its simultaneous electrochemical interactions with aluminum and graphite in the NaOH electrolyte. This finding would shed light on the packing and the structural design of future soft robots owning diverse deformation capability. Moreover, this study related the physical transformation of a non-living LM droplet to the life behavior of amoeba in nature, which is inspiring in human’s pursuit of advanced biomimetic machine.

## Introduction

The cognition and imitation of the unique movements of the biological system has always been the core pursuit for scientists from diverse fields such as physics^1–5^, biology^6–8^ and engineering^8–12^ etc. Artificial machines can hardly achieve functions as elaborate as those achieved by living species. Even the animals with the simplest structure display behaviors that often appear too complicated to be fully imitated. For example, the amoeba, a single-cellular organism, can extend its temporary structures called as pseudopodia in order to move and feed^6^. Its whole body may change rapidly along with the extending pseudopodia. Such free and deformable pseudopodia movements of the amoeba require fine coordination of the cytoskeleton (microfilament) under complicated mechanism^13^, which is still challenging for any artificial machine to achieve.

Despite its complexity, the amoeba-like behavior recently has attracted great attention in the investigation and development of flexible and soft devices^14–16^. Two strategies are often adopted for the design of amoeba-like machines or actuators with good deformability and adaptability. The first one is to connect several modular units together so as to construct the deformable structure^17–20^. However, the deformability of such systems is usually restricted by their physical structures. It is for this reason that the amoeba-like pseudopodia are rarely achieved so far. The other approaches just directly adopt flexible or stretchable sensing materials as actuators^21–24^. Although these actuators to some extent own the deformable body and even amoeba-like pseudopodia, their deformation relies heavily on the external driving forces, which significantly reduces the flexibilities of the machine in the free space.

In this study, diverse morphological transformations of liquid metal machine made of gallium alloy GaInSn (LM for short) with striking external resemblance to amoeba were demonstrated. The basic behaviors take place when the LM droplet was amalgamated with certain amount of Al (LM-Al droplet for short) and then was placed on a graphite substrate immersed in the alkaline electrolyte (0.5 mol/L NaOH in current study). Within this multi-material system, the soft and deformable LM droplet behaves like an amoeba body. Clearly, such LM amoeba activities were brought about by the integrated physical and electrochemical interactions between the components of such multi-material system. Further, as it was revealed, the behaviors of the LM-Al droplet would display even more complex responses than that of the real biological amoeba in nature. The details are clarified as follows.

## Results

**Figure 1:**
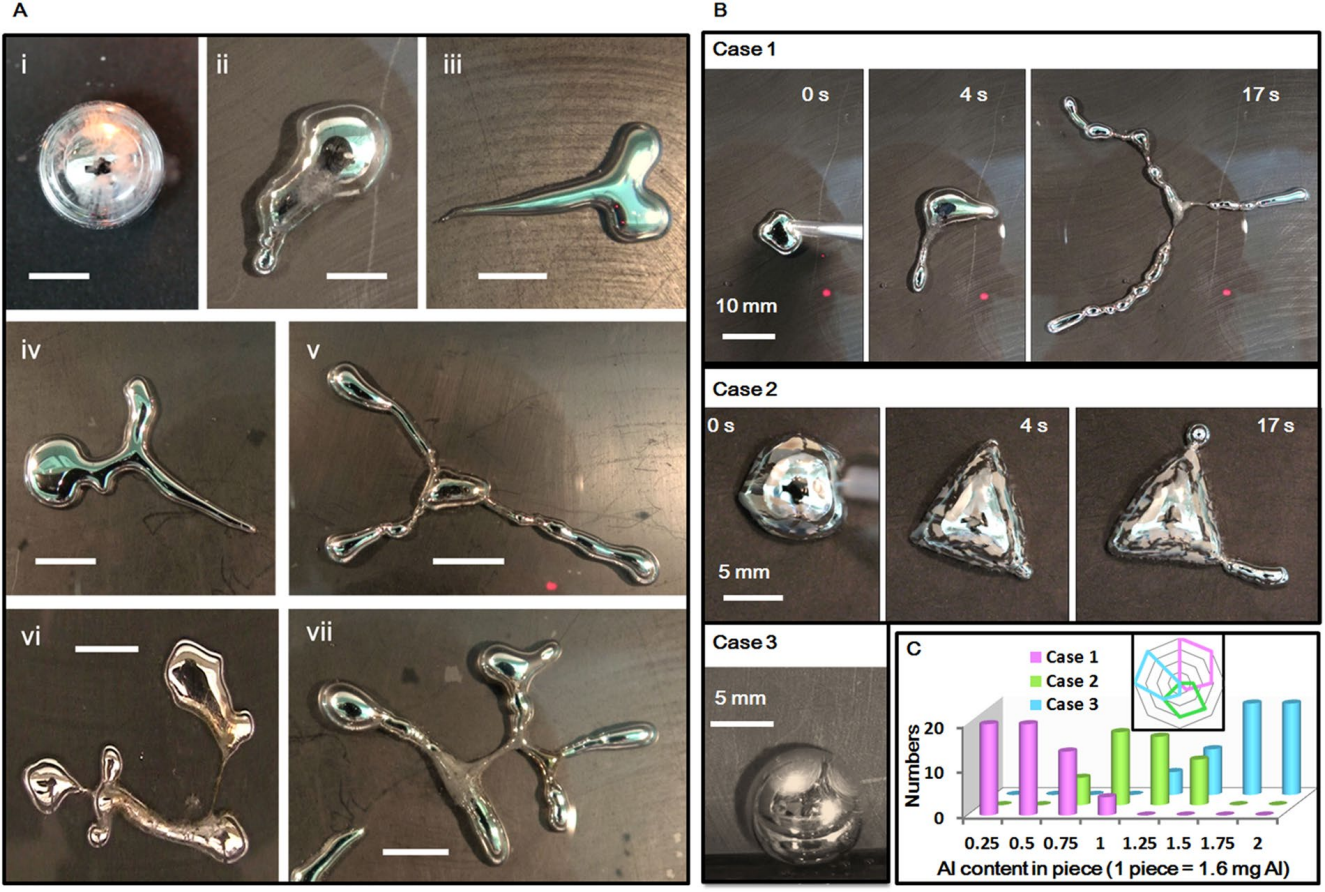
(**A**) i. The LM-Al droplet prepared in NaOH in glass petri dish. ii–vii. Amoeba-like transformations of LM-Al droplets on the graphite. The LM amoeba presents one main “pseudopodia” in ii, three in iii and iv, four in vi and five in vii. Scale bars: i and ii, 3 mm; iii and iv, 5 mm; v, 1 cm; vi and vii, 6 mm. (**B**) Consecutive snapshots of three typical heteromorphous transformation of LM-Al droplet in Case 1 to Case 3. (**C**) The LM droplets with various Al contents presenting different behaviors were categorized and numbered (20 trials for each content. For Al content, 1 piece = 1.6 mg). Inset: The radar plot of the data adapted from Fig. 1C.

Figure 1A exemplified the most typical autonomous amoeba-like morphological transformations of the LM-Al droplet. The LM amoebas with different numbers of main extensions were also observed, which display large resemblance to the pseudopodia. For the purpose of better illustration, these extensions were called LM pseudopodia in the following sections. At this stage, three different types for the morphological transformations of the LM-Al droplets were discovered, which can be categorized as Case 1 to Case 3 based on their distinct properties in appearances (Fig. 1B, Supplementary Movies S1–S3). In Case 1 in which the LM had the lowest Al content, the round LM-Al droplet quickly spread out with the formation of pseudopodia as soon as it contacted the graphite in NaOH. A small number of bubbles were observed on the LM-Al droplet surface during this transformation. In Case 2 in which the LM had relatively higher Al content, we observed that the bright shining droplet quickly became grey and gloomy upon contact with the graphite. Subsequently the round LM droplet deformed and the grey surface continuously cracked. Further, the LM pseudopodia were also observed. Along with these transformations, abundant bubbles were observed on the LM surface. In Case 3 in which the LM droplet had the highest Al content, the LM-Al droplet moved smoothly similarly as it did on the glass substrate. Vortices were observed on the droplet surface. The behaviors of the LM-Al droplet in Case 1 and 2 presented remarkable resemblance to those of amoeba.

Regarding the LM droplets containing varied Al content presented different behaviors, the relation between Al content and droplet behaviors were characterized and quantified (Fig. 1C). Generally, when the Al content was relatively low, the LM-Al droplet would behave as Case 1 described. With increase of the Al content, the droplet behaviors became more likely to be categorized into Case 2. When the LM droplet absorbed enough Al, it would present the behavior in Case 3. There may have certain overlaps in this categorization, which should be due to small variations of different graphite substrates. The radar plot (Fig. 1C inset) presented obvious mutual distinctions among these behaviors of the three Cases based on Al content variations. It indicates that the Al content in the LM should play an important role in the classification of these three categories.

The diverse transformation over this study refers to a complex multi-material system including LM, Al, graphite and the alkaline electrolyte surrounding them. The general interactions among these components are crucial to understand these complicated behaviors. It has been revealed that the bouncing spherical LM gallium alloy droplet can quickly spread and become flat when placed on the graphite substrate immersed in the NaOH electrolyte^25^. The spreading behavior of the gallium through applying potential was once attributed to its electrocapillarity^26^. Recent study suggests that it is the electrochemically induced surface oxide that works as surfactant and reduces the surface tension of the gallium droplet^27^. In our study, a thin layer of oxide was observed on the pure LM surface upon its contact with the graphite substrate in 0.5 mol/L NaOH ($Ga-3{\rm{e}}=G{a}^{3+}$, $G{a}^{3+}$ easily became gallium oxide in the presence of air), which also reduced the droplet surface tension significantly (Supplementary Fig. S1–S3)^25^. Although the NaOH owns the ability to remove the surface oxide $(G{a}_{2}{O}_{3}+2O{H}^{-}+3{H}_{2}O=2Ga{[{\rm{O}}{\rm{H}}]}_{4}^{-})$ ^28^, the surface oxide is still clearly visible on the LM droplet and the flat shape of the pure LM droplet can generally be maintained for at least 30 min. This suggests the oxidative effect of graphite seems stronger than the surface oxide-removing capability of NaOH.

**Figure 2:**
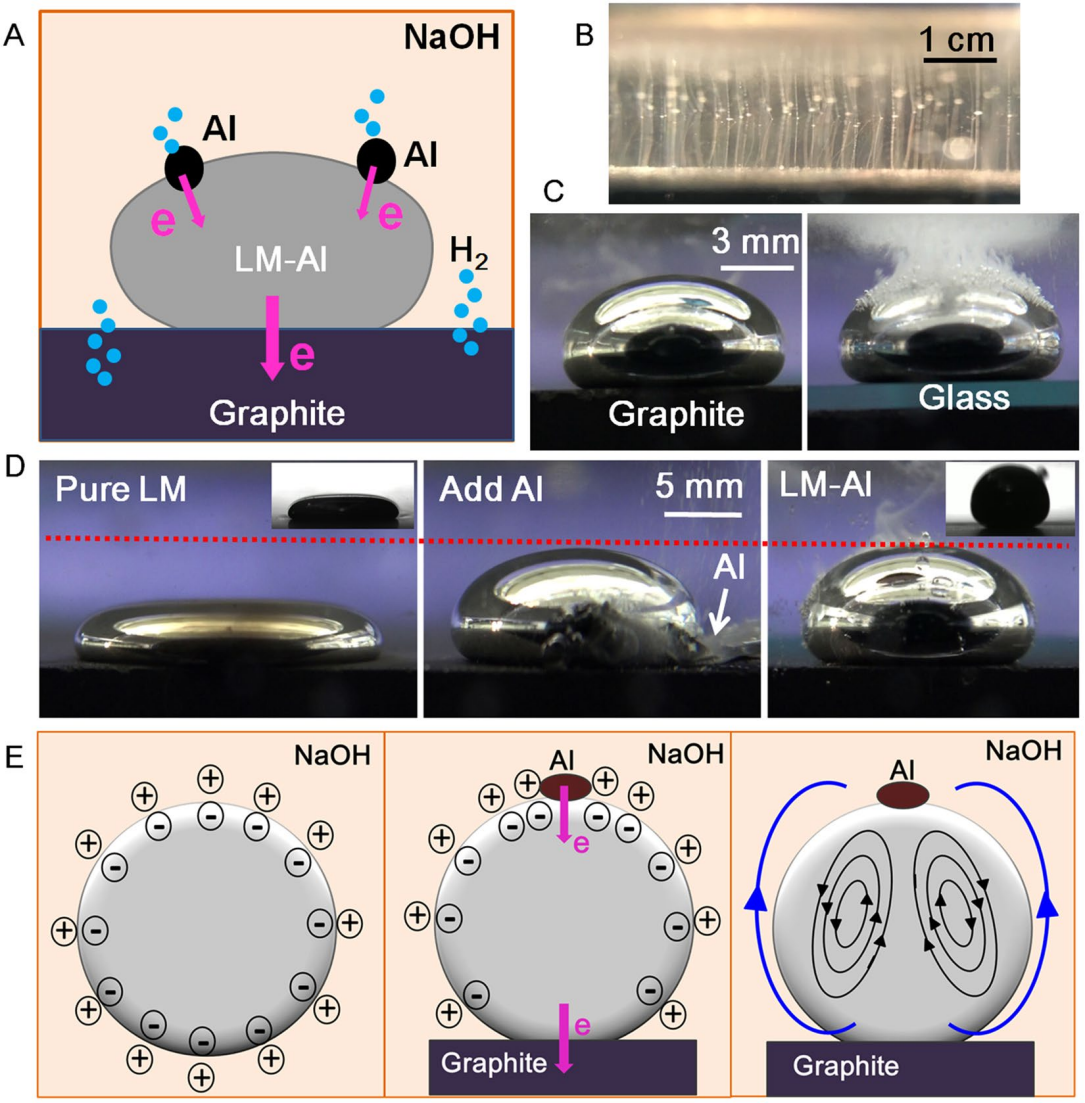
(**A**) Schematic diagram of the electrochemical interactions among Al, LM and graphite. (**B**) Hydrogen was bubbling from the gap of graphite surface. (**C**) Same LM droplet containing high Al in Case 3 on the graphite (left) and glass (right) substrate respectively. Abundant bubbles were observed on the LM-Al droplet on the glass substrate while few were observed on the graphite substrate. (**D**) Al reaction increased surface tension of LM droplet (500 uL) on graphite substrate. Inset: small drop (30–50 uL) of LM (left) and LM-Al (right) on graphite surface for the surface tension measurement. (**E**) The surface tension gradient on LM-Al droplet induced by the electrochemical interactions with Al and graphite. Left: The original surface charge distribution of LM droplet without Al in NaOH. Middle: The Al reaction and graphite interaction induced the surface charge imbalance across the LM-Al droplet. Right: Surface vortices were observed on the LM-Al droplet. The small black arrow rings here indicate the surface vortices directions and the two large blue arrows indicate the surface tension gradient from high to low across the droplet.

The interaction between the LM droplet and the Al in the NaOH has been revealed in previous reports^28, 29^. Briefly, Al can disperse into the LM and form galvanic cells with the LM, which influences the charge distribution over the droplet. Meanwhile, hydrogen is also produced on the droplet. When those components were incorporated together, it was predicted that the Al on the LM droplet would form galvanic cell with the graphite substrate through the LM, since there were potential differences among those conductive materials (the standard electrode potential of Al, Ga and water in alkaline solution are −2.33 V, −1.219 V and −0.8277 V respectively^30^) (Fig. 2A). According to the potential difference of these materials, it is deduced that the Al in the LM-Al droplet should initially react and lose electrons ($Al+4O{H}^{-}-3{\rm{e}}=Al{{O}_{2}}^{-}+2{H}_{2}O$). Then electrons just outflowed to the graphite through the LM. Thus the surface oxide on the LM can be reduced in some extent ($G{a}^{3+}+3{\rm{e}}=Ga$). At the anode (graphite), water should receive those electrons and hydrogen is produced ($2{H}_{2}O+2{\rm{e}}={H}_{2}\uparrow +2O{H}^{-}$). Bubbles were observed to come out from the graphite gaps at least 5 minutes later. This observation supports the claim of galvanic interaction taking place (Fig. 2B). The bubbles did not appear immediately because graphite could store hydrogen^31^. Moreover, when the LM-Al droplet was separated from the graphite, many more bubbles could be observed on the droplet (Fig. 2C, Supplementary Movie S4), which further verified that galvanic interaction between the Al and the graphite substrate in the NaOH solution.

To further investigate and explain these transformations in a more comprehensive and logical way, the phenomenon in Case 3 was examined in detail. Unlike the pure LM droplet without Al which became flat on the graphite substrate, the LM-Al droplet in Case 3 generally maintains the round shape similarly as it did on the insulated substrate (Fig. 2C). The bouncing spherical appearance of the LM-Al droplet with the metallic luster strongly implied that the surface oxide was removed. It has been reported that the Al reaction in the LM droplet can remove the surface oxide over the LM droplet in the NaOH solution and increase the surface tension of the LM^28^. To further demonstrate that the Al reaction would affect the surface tension of the LM-Al droplet on the graphite surface, a comparative experiment was carried out. A 500 μl pure LM droplet without any Al was placed on the graphite initially, which should have good contact with the graphite substrate. Thin oxide layer was formed on the droplet surface, which as a result reduced its surface tension significantly. Then a piece of Al flake (around 2 mg) was added into the flattened droplet via forceps. As the Al was absorbed gradually, the flattened droplet continuously and obviously contracted and became more spherical (Fig. 2D). The contact angles also increased from 119.4 ± 2.3 to 145.2 ± 1.8 degree in this experiment (the average value of contact angles at both sides, n = 5), which suggests that the surface tension significantly increased (p < 0.001). To further quantify the surface tension changes, the surface tension of the LM droplet and the LM-Al droplet on the graphite substrate in NaOH were measured via a goniometer based on the sessile drop method. The surface tension of the LM droplet on the graphite is obtained around 84.3 ± 21.3 mJ/m^2^ (n = 3) (Fig. 2D, left inset). When enough Al was added (5 pieces Al for 500 uL LM), the LM-Al droplet kept rolling on the graphite surface, which made it difficult to define the interfacial interaction between droplet and substrate. However, from the recorded snapshot (Fig. 2D, right inset), the surface tension of the LM-Al droplet can be also estimated as around 387.3 ± 32.6 mJ/m^2^ (n = 3), which is close to that on the glass substrate^27^. Besides, as the Al reaction continued, the surface oxide gradually disappeared based on our observation (Fig. 2D). The significant increase of surface tension of the LM droplet on graphite surface (from 84.3 ± 21.3 mJ/m^2^ to 387.3 ± 3^2.^6 mJ/m^2^, n = 3, p < 0.001) as well as the disappearance of surface oxide in this comparative experiment confirmed that the Al reaction could remove the surface oxide on the LM droplet and restore its surface tension even on the graphite surface. For the LM-Al droplet in the Case 3, as the Al content was relatively high, the electrochemical reduction by the Al reaction was remarkably intensive, which can strongly affect the surface tension due to inhibiting the formation of oxide layer. Thus the LM-Al droplet can maintain its round shape. When the Al continued to be consumed, the droplet would stop moving. And dark oxide membrane quickly appeared on the surface (Supplementary Movie S5). This observation validates that the graphite still plays its role in the formation of oxide layer.

It is noteworthy that obvious surface vortices were observed with Al granules aggregating on the droplet surface especially when the droplet stood a little leaned against the insulated wall of the container (Fig. 1B, Supplementary Movie S3). The angular velocity could be 19.6 ± 3.8 rad/s (n = 3) or even higher (Supplementary Fig. S4). The generation of the vortices should be related to the imbalanced pressure difference between the electrolyte and the LM generated through the electrochemically or electrically induced potential gradients across the electric double layer (EDL) of the LM droplet^28, 32–34^. In this case, as there was no obvious surface oxide on the droplet, it should be the surface charge rather than the surface oxide that plays a more crucial role in determining the surface tension of the droplet. The LM droplet without Al was originally round with negative charge generally evenly distributed on the surface (Fig. 2E, left). When enough Al was dispersed into the LM droplet and LM-Al was placed on the graphite, Al granules were observed to aggregate on the top of the droplet. As the electrochemical reaction of Al went on, the electrons flowed internally from Al to the LM droplet, making more negative charges gather on the top^28^. At the bottom, the electron flowed from LM to graphite, leaving less negative charges, which enhanced the surface charge imbalance (Fig. 2E, middle). As the voltage of the EDL increased at the top of the droplet surface, the surface tension should decrease based on the Lippmann’s equation (${\rm{\gamma }}={\gamma }_{0}-\frac{1}{2}C{U}^{2}$, where *γ*_0_ is the original surface tension of the liquid; *C* and *U* is the capacitance and voltage across the ELD). Similarly, the surface tension should decrease at the bottom. Thus the surface tension gradient was generated across the droplet. The surface tension gradient could induce surface vortices due to the Marangoni effects (Fig. 2E, right), which were also observed in other studies^28, 32, 33^. In this study, apart from the electrochemical reaction of the Al, the electrochemical oxidation by the graphite also contributed to the surface potential imbalance across the EDL of the droplet. As a result, intense surface flows as vortices were observed.

From the above discussion, it reveals that the status of the LM-Al droplet on the graphite in this study was determined by two main factors: The electrochemical oxidative effect via the interaction with the graphite took the electrons from LM and even generate the oxide layer on droplet surface in the presence of air ($Ga-3{\rm{e}}=G{a}^{3+}$); The electrochemical reductive effect from Al reaction transferred electrons to LM and even removed the surface oxide of the droplet ($Al+4O{H}^{-}-3{\rm{e}}=Al{{O}_{2}}^{-}+2{H}_{2}O$, $G{a}^{3+}+3{\rm{e}}=Ga$). Those two factors work together as soon as they were connected through galvanic interactions. Although the NaOH owns the ability to remove the surface oxide ($G{a}_{2}{O}_{3}+2O{H}^{-}+3{H}_{2}O=2Ga{[{\rm{OH}}]}_{4}^{-})$, the concentration here as we used is relatively low, which may not be strong enough to influence the surface properties in large extent. In our preliminary study, higher concentration of NaOH (2 mol/L) was also studied. Although the surface oxide can also be observed on the pure LM droplet on graphite surface, the flattened area decreased (500 μL, 19.5 ± 1.2 mm in diameter, n = 3) compared with the same droplet in 0.5 mol/L NaOH (500 μL, 23.2 ± 1.1 mm in diameter, n = 3), indicating that the surface tension became larger (p < 0.1). Moreover, when Al was added, the Al reaction became more intense in a higher concentration of NaOH, which also enhanced the elimination of the surface oxide. Thus the droplet with higher surface tension should become less deformable under a certain force as the interfacial pressure should increase based on the Young-Laplace equation^35^. Accordingly, in our observation, slight transformation of LM-Al droplet took place but the amoeba-like transformations with pseudopodia-like long extensions were rarely observed in the 2 mol/L NaOH solution. Above all, the LM-Al droplet in the 2 mol/L NaOH has larger surface tension on the graphite, which is not likely to have such tremendous deformation as described in our study. In the present article, we are focusing on the amoeba-like transformations of the LM-Al droplet, which is more likely to take place in NaOH at low concentrations. So we adopted 0.5 mol/L NaOH throughout the test based on our preliminary experimental results.

**Figure 3:**
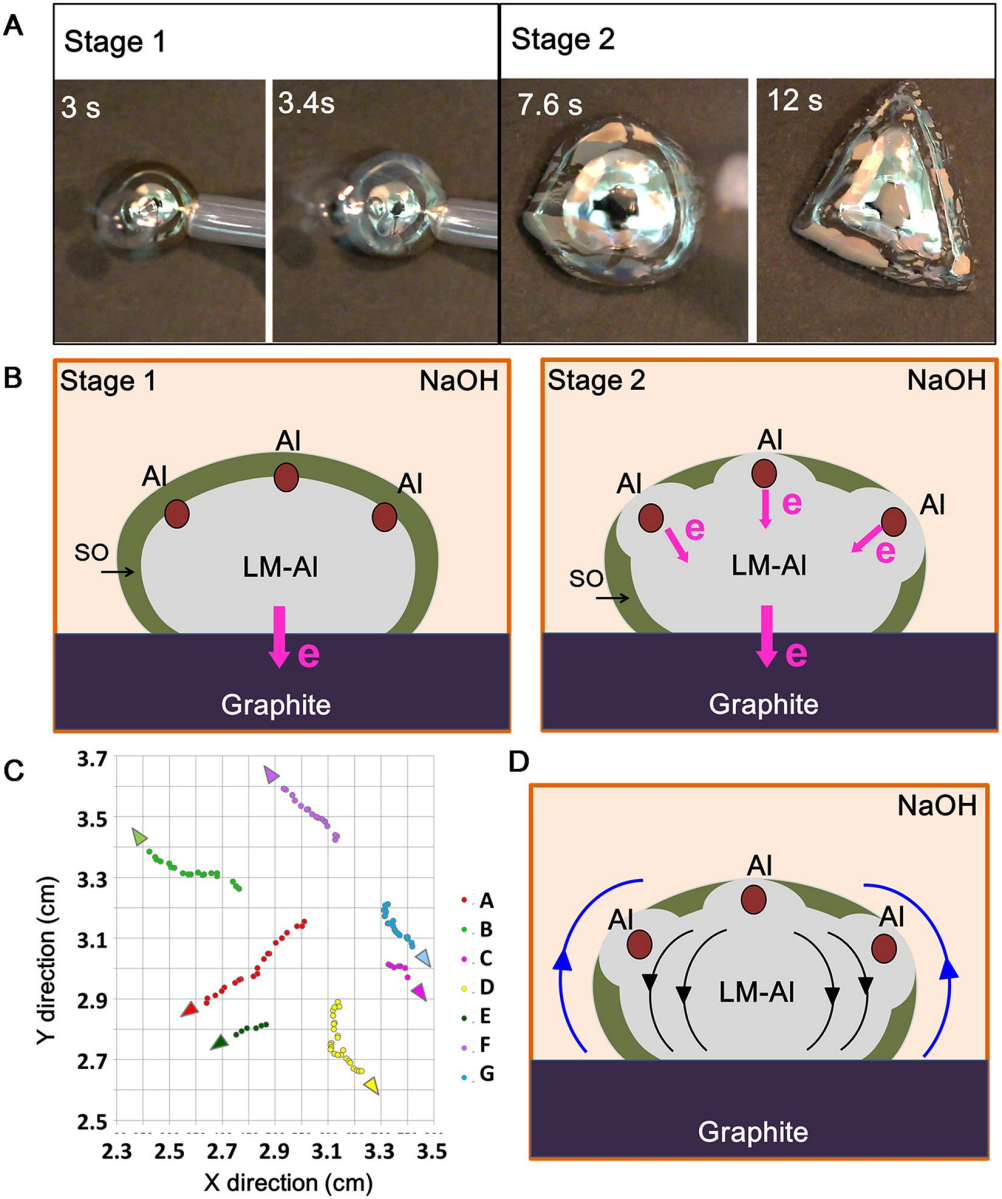
(**A**) The transformation of LM-Al droplet in Stage 1 and 2 in Case 2. (**B**) The schematic diagrams of the electrochemical status of LM-Al droplet in stage 1 and 2. The red dots indicate the evenly dispersed Al on the surface. The dark green periphery round region indicates the surface oxide (SO). (**C**) The trajectory of oxide cracks in Stage 2. The arrows indicate the moving directions of cracks. Each point was taken every 0.2 s. The points were traced at each frame of the video (2D) without concerning the 3D surface. (**D**) The surface out-dispersing of the LM-Al droplet. Black arrows indicate the surface flow directions. Blue arrows indicate the surface tension gradient from high to low across the droplet.

Compared with that in Case 3, in Case 2 the LM droplet with less Al content presented completely different behaviors, giving striking external resemblance to the amoeba movement. In this Case, Al was generally evenly dispersed in the LM droplet without big granules on the surface. Three stages were divided based on the observational properties in order to better explain the amoeba-like behavior including the body deformation and pseudopodia extension, respectively. In the first stage (Stage 1), the round shining LM-Al droplet quickly turned grey pebble upon contact with the graphite in less than 1 second (Fig. 3A), suggesting that quick electrochemical oxidation by the graphite dominated the LM droplet. Unlike the thin oxide layer of the LM droplet without Al which was dull but still with metallic color, the oxidized layer on the LM-Al droplet became dark and grey with no metallic luster, suggesting that the Al oxide may be included in this oxidized metal layer. Besides, bubbles were mostly observed on the oxide fragments but not on the silver-white LM body (Supplementary Fig. S5), implying that there was certain Al in the oxidized membrane and few Al in the newly exposed LM.

Right after the oxide membrane covering the whole droplet, local deformations took place and the round shape of LM-droplet changed to an irregular one like the amoeba body (Fig. 3A, Stage 2). The oxide membrane was observed to break out and newly exposed LM seemed gush out like the boiling water (Supplementary Fig. S6, Movie S7). This should be caused due to reductive effects of Al which removed the oxide layer. At the same time, the electrochemical interaction with graphite continuously oxidized the newly exposed LM, significantly reducing the surface tension. Thus it was observed that the oxide surface on the top continuously cracked and dispersed to the bottom as the reactions went on (Supplementary Fig S7, Movies S2 and S6). We located a certain point (a certain pixel) of the oxide fragment (usually a corner) by its coordinates in Matlab and traced it at each frame of the video (2D images). The trajectory of the oxide fragments also confirmed this out-dispersing flow (Fig. 3C). The surface flow of the droplet should be driven by the surface tension gradient across the droplet, which is also an analogy of the Marangoni effects. As the liquid with lower surface tension should flow to the higher region, it suggested that the surface tension at the top was lower than that close to the bottom (Fig. 3D).

**Figure 4:**
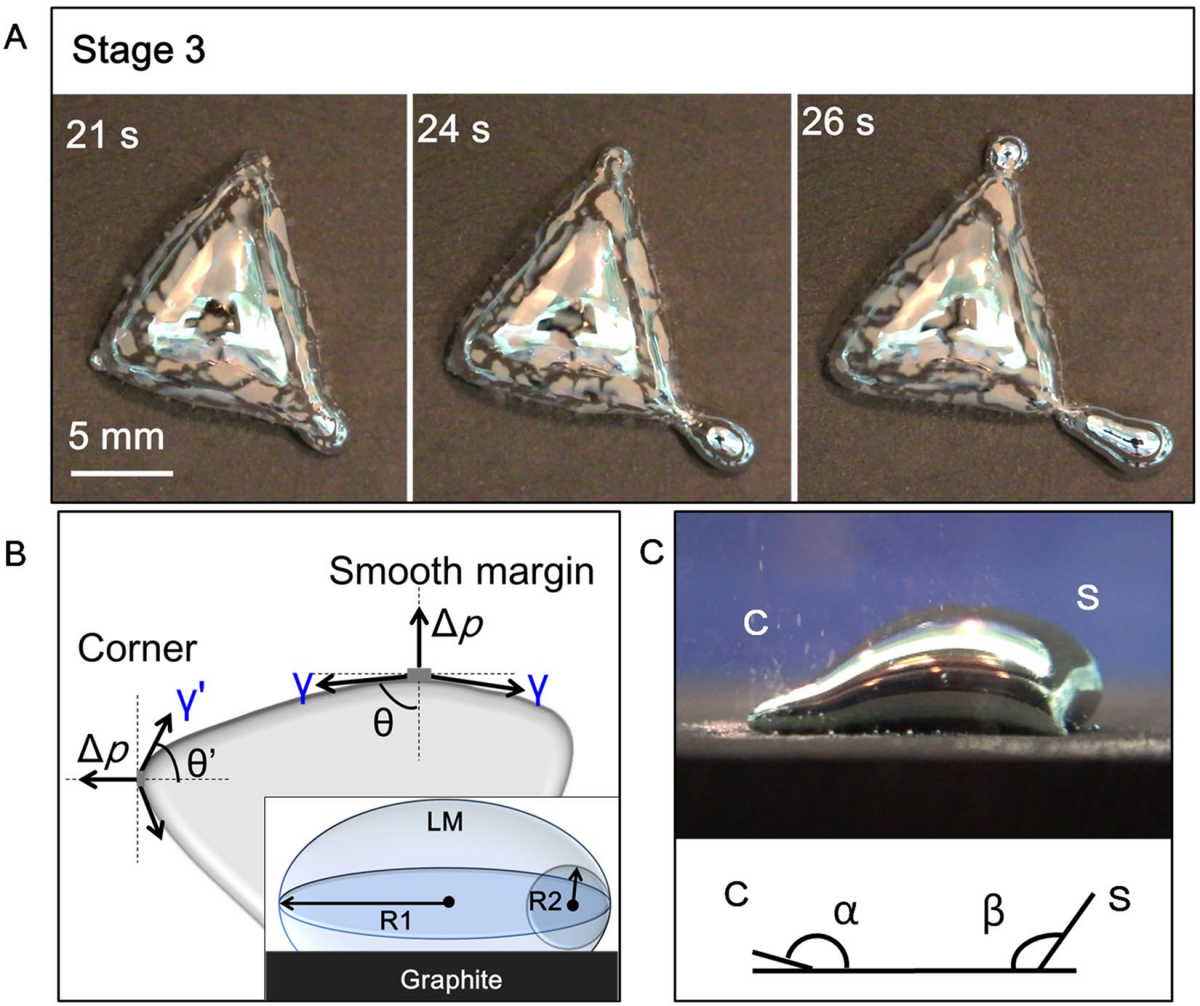
(**A**) The consecutive snapshots of the stretching out LM pseudopodia. (**B**) The force analysis indicates that the surface tension at the corner was lower than that at the smooth margin in the top view. The inset indicates the R1 and R2 in a LM droplet on graphite substrate in the horizontal view. (**C**) The side-view image of a LM-Al droplet with pseudopodia. C: Corner; S: Smooth margin. The contact angle at the corner (α) was observed smaller than that (β) at the smooth margin.

In the third stage (Stage 3), slender LM pseudopodia gradually stretched out from the LM amoeba body (Fig. 4A). According to the discussion for Case 2, the surface tension gradient led the LM surface out-dispersing from top to bottom and tucking to the center. Considering the ideal situation in which the Al was evenly distributed on the LM droplet, the droplet should maintain the round shape in the top view on the graphite substrate. The LM surface flowed from top to bottom induced by surface tension gradient. If at all directions the gradient force was equal, the LM droplet should contract to the central and may form convection under certain status (Fig. 3D), which may be an analogy to the Rayleigh-Benard Convection. However, in real situation, the surface tension around the whole droplet was not uniform due to slightly imbalanced distribution of the Al or/and the small contact variations with the graphite substrate (Supplementary Fig. S8)^36^. Thus the surface flow may not be so stable to form convection, and would move along other directions. As the Al continued to be consumed, the surface tension of the whole LM-Al droplet should gradually decline along with Al consumption. Thus the droplet should spread out. As the surface tension around the droplet is not uniform, the droplet should be more likely to spread out at those points where the surface tension is lower. In this way, the droplet just became deformed.

To further test the speculation that LM droplet should stretch out at the points where surface tension is lower, the force analysis was carried out. Based on the Young-Laplace equation ${\rm{\Delta }}p={\rm{\gamma }}/(\frac{1}{{R}_{1}}+\frac{1}{{R}_{2}})$ (1/R_1_ and 1/R_2_ are the principal curvatures for the curved surface at periphery of the droplet in the top and horizontal view respectively. Here the droplet is still considered to be a round sessile drop, so R_1_ and R_2_ should be approximately the same at all the points around the periphery of the droplet), if there is a position where the surface tension (γ) is lower, the pressure difference between the LM and electrolyte (Δ*p*) is also lower. Thus the LM at higher pressure difference should propel the LM out from the points where the pressure difference was relatively lower, which leads to the initiation of the pseudopodia. As a result, our speculation was confirmed.

After the LM-Al droplet deformed to form angles or corners at those points where the surface tension was lower, it continuously stretched out to form long extensions like pseudopodia (Fig. 4A). It was speculated that the surface tension of the points where the corners was still lower than that at the smooth margin. Thus the LM could continuously extend out to form the long pseudopodia. Based on this speculation, the force analysis at the LM-reach-out points was made for the investigation of further extension of the pseudopodia. According to the Young-Laplace equation, the interfacial tension of the curved surface at one side is

1 $${\rm{\gamma }}={\rm{\Delta }}p\,/(\frac{1}{{R}_{1}}+\frac{1}{{R}_{2}}),$$

where Δ*p* is the pressure difference between the LM droplet and the electrolyte (Δ*p* > 0), 1/R_1_ and 1/R_2_ are the principal curvatures for the curved surface as illustrated in Fig. 4B inset. R_1_ is perpendicular to R_2_. In the side view, R_2_ should maintain approximately the same at the points around the periphery. However, R_1_ depends much on the shape of the droplet from the top view. For the deformed droplet in Fig. 4B, $\frac{1}{{R}_{1}}=cos\theta$. Thus the interfacial tension can be deduced as

2 $${\rm{\gamma }}={\rm{\Delta }}p\,/(\frac{1}{{R}_{2}}+\,\cos \,\theta ),$$

In the quasi-steady state, Δ*p* (Δ*p* > 0) and R_2_ are considered equal at every hemisphere. At the smooth margin, *θ* is obviously larger than that at the corner (*θ*′). Therefore $\cos \,\theta  < \cos \,\theta ^{\prime}$ and γ > γ′. Thus it is deduced that the interfacial tension at the smooth margin (γ) is larger than that at the corner (γ′). In other words, the surface tension at the corner was lower. Thus the LM was further stretched out at those deformed corners, which further formed the slender and long pseudopodia. The side-view of a LM-Al droplet with pseudopodia showed that the contact angle between the droplet and the substrate at the pseudopodia-stretched-out corner is obviously smaller than that at the smooth margin (Fig. 4C), validating that the surface tension was lower at the corner. Thus it was proven that the LM was more likely to further extend from the points at the corner to form longer pseudopodia.

Based on the above discussion, the phenomenon in Case 1 now became clear. In Case 1, the Al content was much lower in the droplet. Thus the grey oxide layer containing much Al oxide was not observed in Case 1. Moreover, the surface tension of the droplet should be generally lower than that in the Case 2, rendering more deformability of the droplet. Thus it was observed that the LM-Al droplet directly spread out upon contacting with the graphite substrate (Fig. 1A, Case 1) without experiencing the Stage 2 in Case 2. The LM pseudopodia formation should share similar mechanism with that in Case 2. As a whole, the contact angles of the droplet in Case 3 (around 137 degree, data measured from Fig. 2C) are larger than that in Case 1 or 2 (pseudopodia side: 96 degree; smooth margin side: 136 degree, data measured from Fig. 4C), which is consistent with our conclusion that the surface tension of the droplet in Case 3 is generally larger than that in Case 2 and Case 1.

In these heteromorphous transformations of the LM-Al droplets, the reductive effect by Al reaction and the oxidative effect by graphite were two crucial factors in determining the LM-Al droplet behaviors as described above. Both factors competed and collaborated together during the whole transformation processes, which generated the surface tension gradient and induced such intriguing and abundant transformational behaviors of LM droplet. The LM droplet in our study had apparently much larger deformation than those caused by other factors such as ionic^37^ or surface charge imbalance^28, 32^. The deformation was totally self-powered, which is quite different from the previous gallium deformation induced by directly applying potential^27, 38, 39^. Besides, it was recently found that^40^, liquid metal can even be used as an anode to drive redox reaction which would lead to spontaneous growth of the hydrophilic gallium oxide and then induce LM flow into a channel. Unlike this interesting LM extensions shaping by channels, the present transformations are completely automatic in free space, which is useful for future soft device design. The phenomena are based on the aluminum powered liquid gallium alloy machine and its unique interactions with graphite immersed in the alkaline solution. It is the multi-materials system that renders the soft LM droplet’s diverse transformations, which may be capable of performing some functions such as actuator, switch or trigger in soft devices. Apart from the above integral factors, there are other factors which may influence the transformation significantly. For example, if there were big Al granules aggregating on part region of the droplet, the bipolar reaction of the Al would strongly influence the transformation of the droplets (Supplementary Fig. S9). Besides, as the graphite was a loose and porous material, the roughness and surface topography may vary at different places (Supplementary Fig. S8), which should also influence the contact angles of the LM droplet on the graphite and in turn their surface tension at local positions^36^. This finding also suggests the self-adaptive property of such metal amoeba, which has practical values in developing potential soft robots for complex environment^41^. Since the completion of this previous project we continuously work on the examination of other possible LM transformations on substrate materials. The additional findings^42^ further strengthen the claim that the surface charge of the specific substrate material in NaOH solution and the well contact between droplet and substrate are two most important factors in inducing the LM amoeba behavior.

In summary, the intriguing self-fueled amoeba-like transformations of LM-Al droplets were discovered for the first time. The fundamental mechanism underlying these unconventional behaviors has been revealed to be the surface tension variations induced by the cooperative effects of the electrochemical reduction by Al reaction and the electrochemical oxidation via graphite. This finding, which closely resembles the amoeba biology in nature, would promote peoples’ basic understanding of the soft characteristics of liquid gallium alloy machine fueled by Al. Moreover, the multifunctional behaviors of such hybrid material system also offer new insights for the design of future soft devices even bionic robots which would own large and diverse shape transformation capability.

## Methods

### LM-Al droplet transformation on the graphite surface

All the liquid metal (LM) droplets used in the experiments were GaInSn alloys prepared from gallium, indium and tin with purity of 99.99%. These raw materials with mass ratios of 67:12:13 respectively were added into a beaker and then heated to 100 °C. A magnetic stirrer was applied to stir the mixture uniformly after the metals were all melted. All the NaOH electrolytes used in current experiments were freshly made at 0.5 mol/L. The graphite plate with purity of 99.9% takes size as 10 cm × 10 cm × 1 cm in length, width and height, respectively.

As the aluminum foil is too light to weight, in our experiments a big piece of aluminum foil was weighted and then cut into several smaller pieces with the same size (1.6 mg = 1 piece) for preparation. A drop of LM was initially injected into the NaOH solution in the glass petri dish. Small piece of Al foil was placed in touch with the LM droplets via forceps. Thus Al was attached to the LM droplet and gradually broken into smaller granules since the gallium would destroy the inter-granular bonds of Al foil and penetrate into the Al grain boundaries^43, 44^. Some of the Al granules were dispersed inside the LM while others accumulated together into big granules on the droplet surface. When the Al foil were dispersed into small granules without apparent big debris, the LM droplets containing Al (LM-Al droplet in short) were gently transferred by a sucker onto the graphite substrate immersed in the NaOH solution. Then the transformations of LM-Al droplet were observed and recorded through digital video equipment (Sony HDR-PJ670). Over the experiments, the graphite surface was ensured to be horizontal to avoid interference from gravity unless otherwise indicated.

To evaluate the relation of Al content with the transformational categories, various amount of the Al foil (1.6 mg = 1 piece) was dispersed in the LM droplets with certain specific volume (500 μL). These LM-Al droplets were then placed on the graphite surface for subsequent observations. For each of the Al content, 20 trials were carried out and their transformations were statistically categorized as shown in Fig. 2B.

### Zeta potential measurement

Alkaline solutions with different pH concentrations were prepared with NaOH and diluted water. The nanoparticles were purchased from DK Nano Technology Co. Beijing. The concentration of each conductive nanoparticle was: 0.15 mg/ml graphite, 0.3 mg/ml copper and 0.4 mg/ml stainless steel, respectively. Then the zeta potential of those nanoparticles was measured by the Beckman Coulter Delsa Nano C Zeta Potential Analyzer (USA).

### Surface tension measurement

In this study, the surface tension generally indicates the interfacial tension between the LM droplet and the surrounding liquids unless otherwise specified. The surface tension was determined via a goniometer (Powereach JC2000D3). Sessile droplets of LM with the volume of 30 to 50 uL were placed on the graphite substrate. Then goniometer determined the volume and the interfacial tension. For the LM-Al droplet, it kept rolling on the surface, which made it difficult to define the interfacial tension between substrate and droplet. So the surface tension of the LM-Al droplet on the graphite was not determined here. Anyway, the surface tension of the rolling LM-Al droplet can be acquired from the recorded snapshots, which can offer some more information about the droplet. All the measurements were carried out at least three times to obtain the average values.

## Electronic supplementary material

Supplementary Information

Movie S1

Movie S2

Movie S3

Movie S4

Movie S5

Movie S6

Movie S7
