# Supplementary material for: Liquid metal amoeba with spontaneous pseudopodia formation and motion capability
Source: Sci Rep. 2017 Aug 3;7:7256. doi: 10.1038/s41598-017-07678-8 (PMC5543163; doi:10.1038/s41598-017-07678-8)
Supplement: Supplementary file 1 — Supplementary Information [file 41598_2017_7678_MOESM1_ESM.pdf]

Supplementary Material

Liquid Metal Amoeba with Spontaneous Pseudopodia Formation and Motion Capabilities

Liang Hu [1], Bing Yuan [1], Jing Liu [1, 2]*

1. Beijing Key Lab of CryoBiomedical Engineering and Key Lab of Cryogenics,

Technical Institute of Physics and Chemistry,

Chinese Academy of Sciences, Beijing, China

2. Department of Biomedical Engineering, School of Medicine,

Tsinghua University, Beijing, China

***Correspondence:** jliu@mail.ipc.ac.cn

Tel. +86-10-82543765

Fax: +86-10-82543767

This file includes:

Figs. S1 to S9

Tables S1

Captions for Movies S1 to S7

Supplementary Figures

The interaction between LM and graphite in alkaline solution

In our latest study, an intriguing and distinct phenomenon was discovered that when the spherical LM droplet was placed on the graphite substrate immersed in the electrolyte, it would easily change to a dull, flat puddle (Fig. S1). The mechanism underlying this flatten deformation is mainly related to the surface tension reduction induced by the formation of a thin oxide layer over the LM surface (Fig. S2)[1]. The quick formation of this oxide layer should be caused by the electrochemical reaction induced by the graphite. It was predicted that the potential drop across the LM-NaOH interface was increased upon the LM-graphite contact. To testify this prediction, zeta potential of the graphite nanoparticle in NaOH was measured. At pH 11.6, the zeta potential of the graphite was -31.6 mV, which indicated that it was a positive potential drop across the graphite-NaOH interface. It is known that the LM droplet is negatively charged in NaOH [2]. Upon contact with the graphite, the negative potential drop across the LM-NaOH interface could be significantly pulled up in order to reach equal potential with the graphite (Fig. S3). Thus our prediction was proven that the LM was electrochemically oxidized, which as a result led to the electrochemical formation of the oxide layer over the LM and reduce the surface tension subsequently.

Further, the zeta potentials of other conductive substrate material including Cu and stainless steel were also measured (Table S1). Similar flattening behavior of the LM droplet was observed on the copper substrate which however should be mainly caused by the spreading of the gallium on copper as it is known that LM gallium alloy

amalgamates copper on the surface [3]. The zeta potential results also confirmed that this flattening is not related to the interface potential changes as the potential drop across the copper-NaOH was negative, similar to that of LM-NaOH interface. While on the stainless steel substrate, such flattening of LM was not observed. This is consistent with the zeta potential result. All these facts further confirmed our prediction that the oxidization of LM was due to its potential elevation pulled by the potential drop across the graphite-NaOH interface.

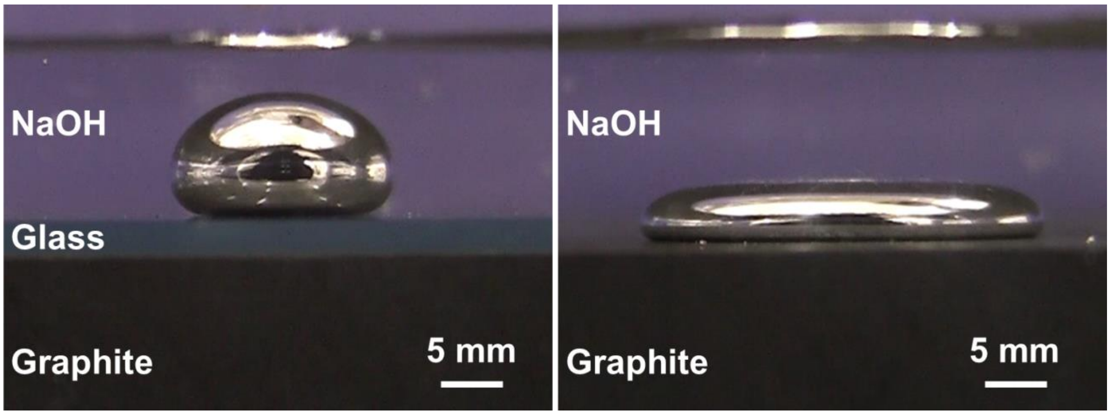

Figure S1. The static images of LM droplet on glass (left) and graphite substrate (right) in NaOH, respectively. LM presented quasi-sphere on glass substrate and became flat on graphite. Data were extracted from our previous published reports.[4]

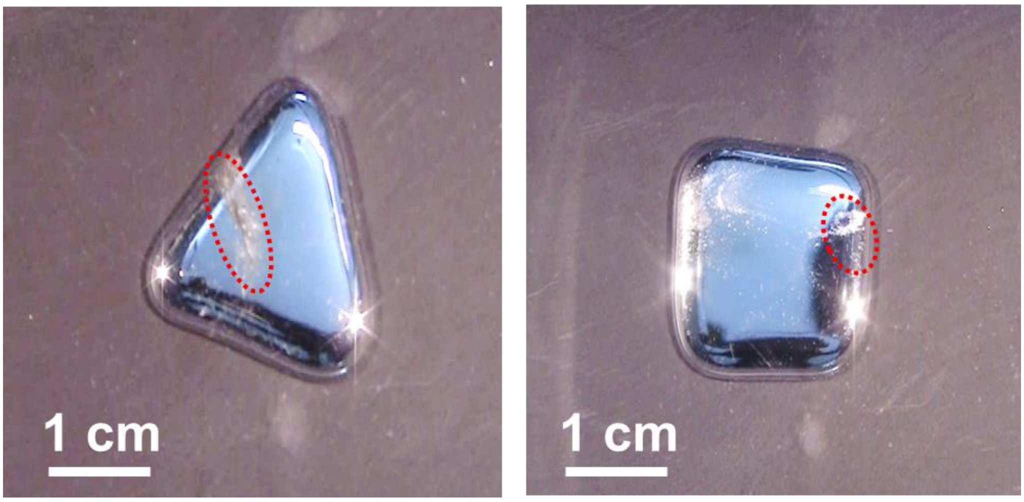

Figure S2. Oxidized membrane was observed on the LM surface indicated by the red dotted circles. Data were extracted from our previous published reports.[4]

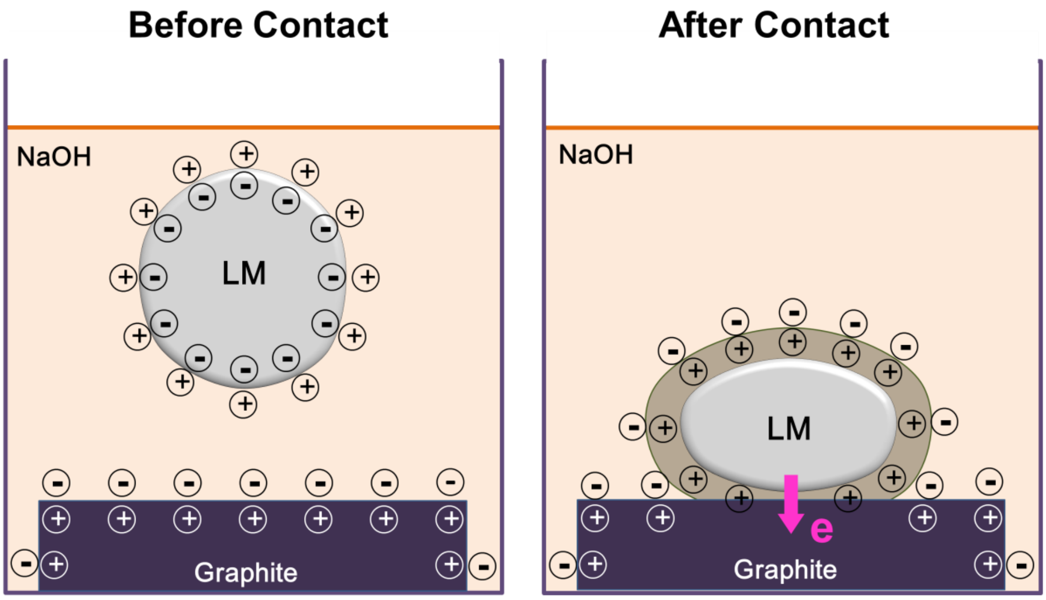

Figure S3. Schematic pictures for the LM transformation before and after contact with the graphite substrate. [4]

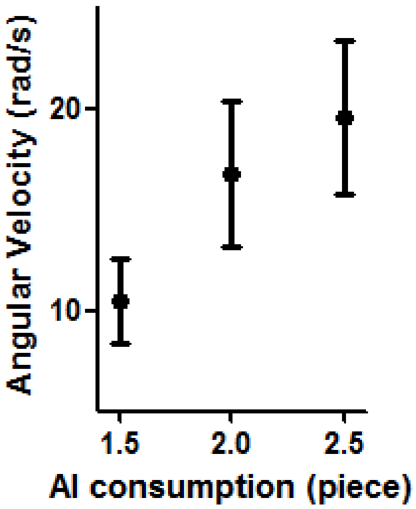

Figure. S4. The relation between Al content and the vortex angular velocity in the LM-Al droplet. The LM-Al droplet was prepared with 500μL LM with various amount of Al (1.6 mg=1 piece). When the graphite was placed horizontal, the droplet kept moving, which made it hard to observe the stable vortices. In this situation, the graphite substrate was a little slant (less than 1 degree) in order to observe stable and intense vortices and the velocity was measured.

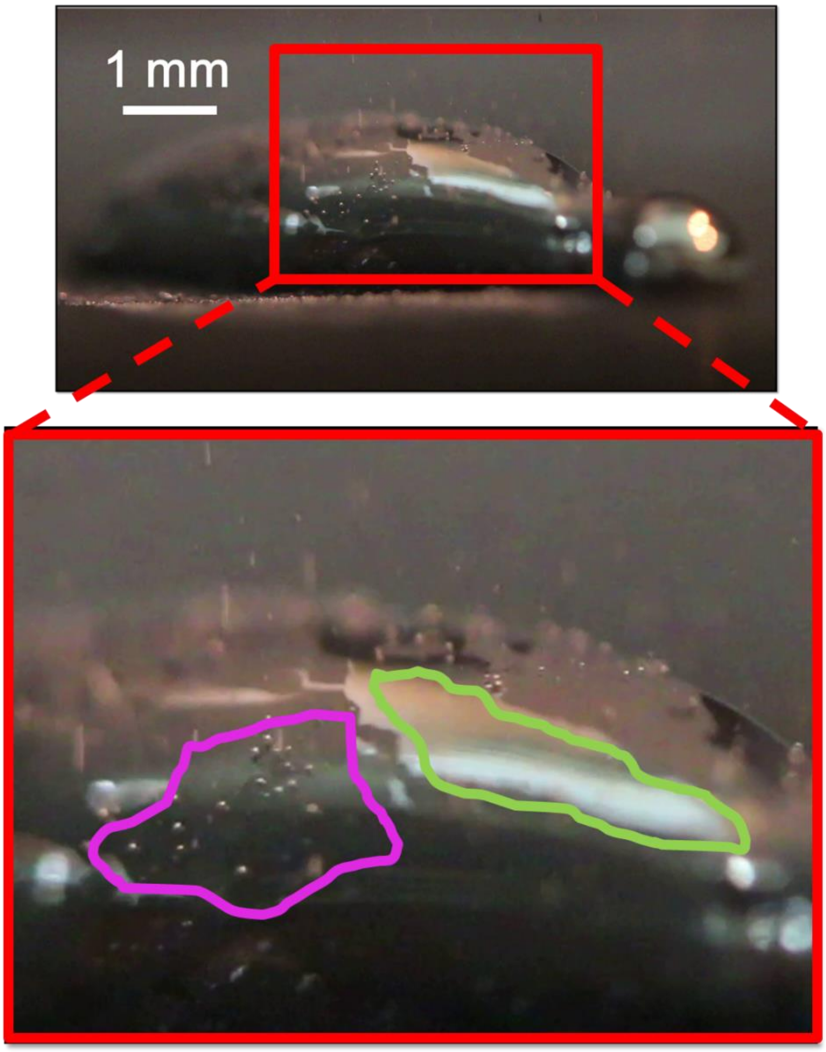

Figure S5. Bubbles were observed on the gloomy oxide layer (pink marked area) but not on the LM body (green area), suggesting that there was Al in the oxide layer.

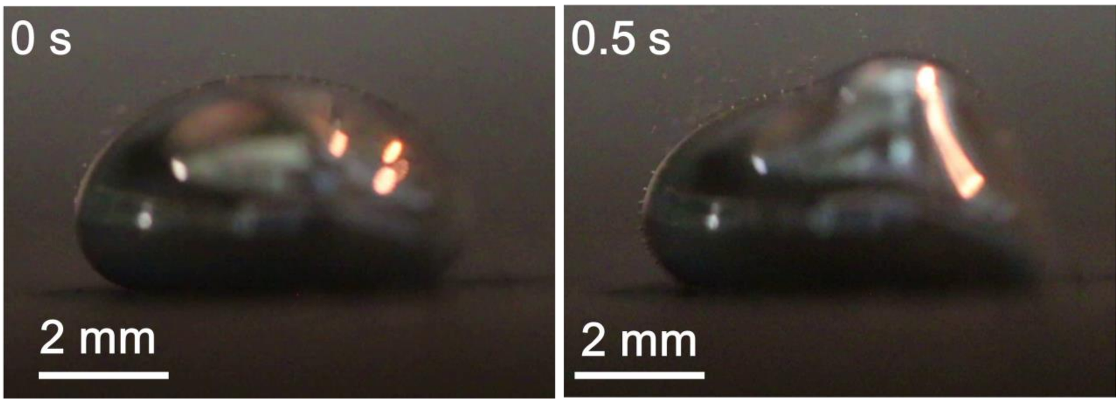

Figure S6. The oxide membrane was observed to break out with LM gush out.

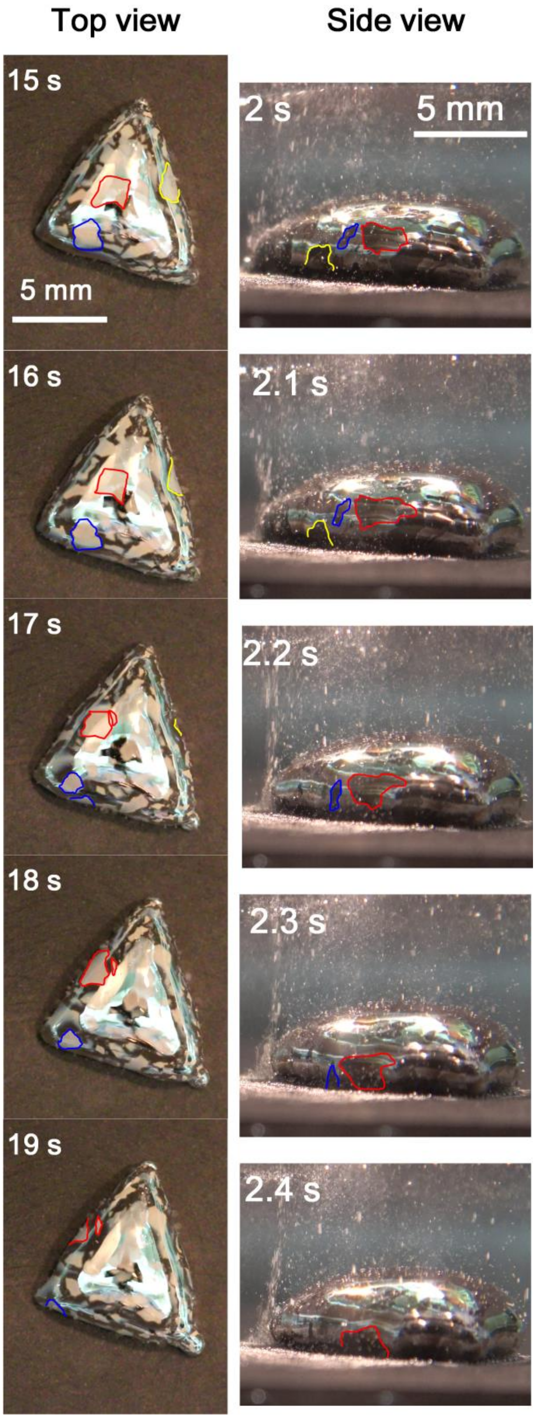

Figure. S7. Consecutive images of the droplet in Case 2. The top surface continuously dispersed to the periphery and rolled to the bottom, tucking to the central bottom. The droplets in the top and side view were different.

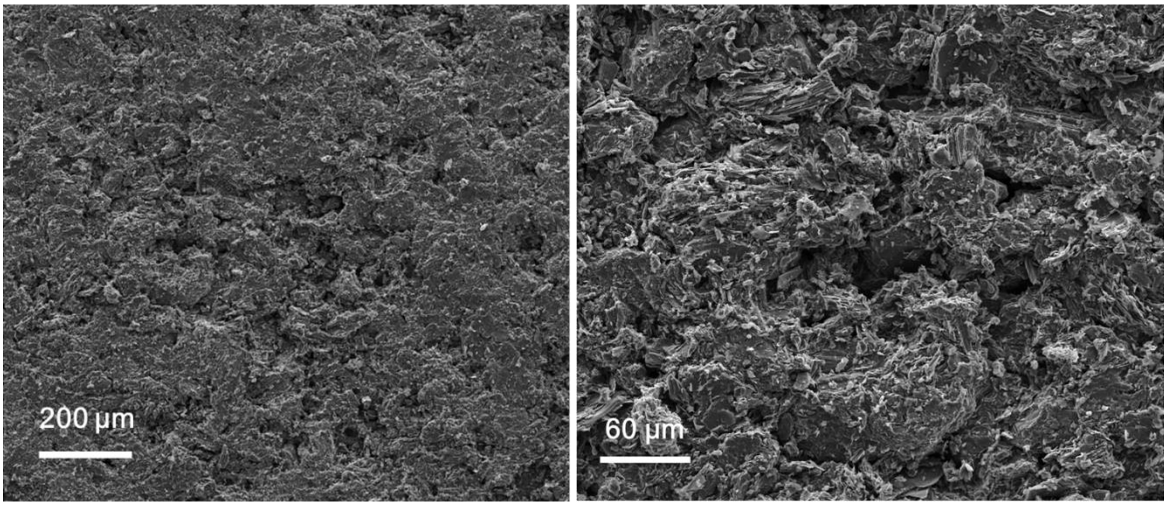

Figure S8. The SEM images of the surfaces of the graphite substrate.

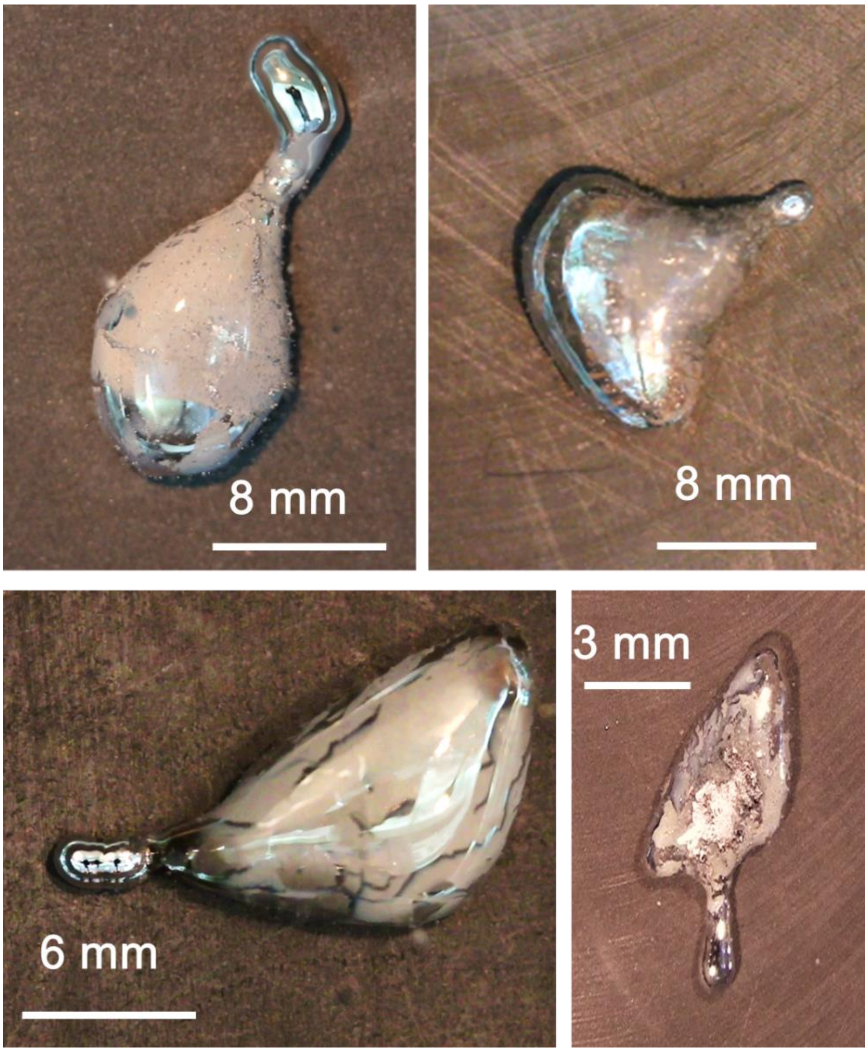

Figure S9. Various morphologies of the LM-Al droplet in Case 2 when the obvious Al aggregated on the droplet.

Supplementary Table

Table S1. Zeta potential of several typical conductive substrate materials in alkaline electrolytes. Unit: mV. Data were extracted from our previous published reports.[4]

pH	Graphite	Gu	Stainless Steel
11.6	-31.6	2.3	0.6

Supplementary Movie

Movie S1

The transformation of the LM-Al droplet in Case 1.

Movie S2

The transformation of the LM-Al droplet in Case 2.

Movie S3

The transformation of the LM-Al droplet in Case 3. The first half showed a LM-Al droplet in Case 3 moving on the graphite. The second half showed the obvious vortices on the LM-Al droplet when the droplet turned a little leaned against the plastic wall of a petri dish.

Movie S4

The LM-Al droplet in NaOH stopped bubbling when contacted with the graphite. When the droplet and graphite were separated (by a sucker), bubbles appeared against on the droplet. This experiment verified the galvanic interaction between Al and graphite substrate in NaOH solution.

Movie S5

When the Al continued to be consumed, the droplet may stop moving around with dark oxide membrane appeared on its surface.

Movie S6

The side view video of the LM-Al droplet in Case 2. The top surface kept rolling to the bottom and tucking to the central.

Movie S7

In Case 2, the oxide membrane was observed to break out with LM gushing out.

References

1 Chrimes, A. F., Berean, K. J., Mitchell, A., Rosengarten, G. & Kalantar-zadeh, K. Controlled electrochemical deformation of liquid-phase gallium. *ACS Applied Materials & Interfaces* **8**, 3833-3839 (2016).

2 Tang, S. Y. *et al.* Liquid metal actuator for inducing chaotic advection. *Advanced Functional Materials* **24**, 5851-5858 (2014).

3 Huttenloch, P., Roehl, K. E. & Czurda, K. Use of copper shavings to remove mercury from contaminated groundwater or wastewater by amalgamation. *Environmental Science & Technology* **37**, 4269-4273 (2003).

4 Hu, L., Wang, L., Ding, Y., Zhan, S. & Liu, J. Manipulation of liquid metal on graphite surface. *Advanced Materials* **28**, 9210–9217 (2016).
